# Routing with Renewable Energy Management in Wireless Sensor Networks

**DOI:** 10.3390/s21134376

**Published:** 2021-06-26

**Authors:** João Junior, Moysés Lima, Leandro Balico, Richard Pazzi, Horácio Oliveira

**Affiliations:** 1 System Technical Group, Sidia Institute of Science and Technology, Manaus 69055-035, Brazil; joao.junior@sidia.com; 2 Institute of Computing, Federal University of Amazonas, Manaus 69080-900, Brazil; 3 Department of Computer Science, National Institute for Amazon Research, Manaus 69067-375, Brazil; moyses.lima@posgrad.inpa.gov.br; 4 Department of Computer Science, Federal University of Roraima, Boa Vista 69310-000, Brazil; leandro.balico@ufrr.br; 5 Faculty of Business and Information Technology, Ontario Tech University (UOIT), Oshawa, ON L1G 0C5, Canada; richard.pazzi@uoit.ca

**Keywords:** opportunistic routing, renewable energy, energy efficiency, wireless sensor networks, routing

## Abstract

In wireless sensor networks (WSNs), power consumption is an important aspect when designing routing protocols. When compared to other components of a sensor node, the power required by radio transmitters is responsible for most of the consumption. One way to optimize energy consumption is by using energy-aware protocols. Such protocols take into consideration the residual energy information (i.e., remaining battery power) when making decisions, providing energy efficiency through the careful management of energy consumption. In this work, we go further and propose a new routing protocol that uses not only the residual energy information, but also the available renewable energy information from renewable energy sources such as solar cells. We then present the Renewable Energy-Based Routing (REBORN) algorithm, an energy-aware geographic routing algorithm, capable of managing both the residual and the available energy. Our results clearly show the advantages and the efficiency achieved by our REBORN algorithm when compared to other proposed energy-aware approaches.

## 1. Introduction

A wireless sensor network (WSN) is composed of several sensor nodes that, together, can monitor and collect data from an area of interest. In many of the envisioned scenarios, these areas have singular features such as high and low ground terrains, lakes, and obstacles, that demand self-organizing routing algorithms [1,2]. Another critical challenge in these networks is how to optimize the energy consumption of the sensor nodes since they can be deployed in inhospitable places with difficult access, making it unfeasible to change batteries [3,4,5].

In most scenarios involving the exchange of information among the sensor nodes, the network lifetime is limited by the energy depletion of the sensor batteries. Thus, several solutions have been proposed in the literature that aim at extending the lifetime of a WSN. For instance, several new routing protocols have been created aiming at scenarios in which energy awareness is the main focus [6,7,8,9].

Among these new routing protocols, the use of greedy forwarding schemes can be considered a common practice [10]. Greedy forwarding uses the known position of the sensor nodes to select the next hop toward the sink node when routing data. This position information can be obtained through the use of GPS devices or by executing a distributed localization algorithm [11]. This routing technique is simple to implement, has low processing and memory requirements, and is extremely self-organizing, since each node only needs local positioning information from its neighbors [12,13]. However, the optimization of energy resources can significantly impact the routing processes in a WSN. Most proposed energy-aware routing protocols use the residual energy of the sensor nodes to provide routes that minimize the number of hops while trying to avoid sensor nodes with almost depleted batteries [14,15].

More recently, solutions based on energy harvesting and renewable energy resources have been proposed. This is an exciting technology for WSNs, since many envisioned scenarios are based on open, outdoor areas in which external energy sources can be found. As shown in [16], sensor nodes can be equipped with devices that can both collect and store energy so it can be used to increase the network lifetime. This energy harvesting can be done, for instance, by using solar cells that are also able to identify the intensity of the available energy, important information that we will explore in our solution.

Thus, in this work, we are proposing a new and novel routing algorithm, called Renewable Energy-Based Routing (REBORN), that can route the gathered information toward the sink node while also managing the energy consumption of the sensor nodes by using the knowledge of both residual energy and available renewable energy. By combining the residual energy information with the available solar intensity (provided by the solar cells), our proposed solution can make better decisions on whether to send the available information, sending extra data when the energy is abundant while reducing the energy consumption when the batteries are low and/or when the available renewable energy is scarce (i.e., no sunlight availability).

Our contributions are summarized as follows:

- We propose a new routing algorithm for WSNs that takes advantage of both the residual battery energy and the available renewable energy to allow energy savings.
- Our proposed algorithm not only generates efficient path routes, but also decides when is the best time for a node to send the data in order to save energy.
- We also propose a new and simplified solar model to be used on the simulation of WNSs with renewable energy.

The remaining of this This paper is organized as follows. In the next section, we describe the related work. Section 3 describes our proposed REBORN algorithm, which is evaluated in Section 4. Finally, Section 5 presents our conclusions and future directions.

## 2. Related Work

Energy-based algorithms in WSNs can be classified into three distinct categories: (1) energy efficiency, (2) energy awareness, and (3) energy harvesting.

In the first category, energy-efficient algorithms try to extend the lifetime of the network by exchanging battery-level information and avoiding the full depletion of sensor nodes. In [17], two routing schemes are proposed to forward data toward the sink node. In this solution, energy efficiency is accomplished by carefully choosing the routes with more available energy, even if it means longer routes. In the Efficient Energy Aware Routing (EEAR) [18], sensor nodes are equipped with GPS (Global Positioning System) to estimate their positions, and this information, with the battery level, is broadcasted by each node to its neighbors so they can make better decisions when choosing the next hop on the routing process.

In the second category (energy-aware algorithms), the sensor nodes have information regarding their battery levels and use this information to increase the network lifetime as a whole [19]. For instance, in the Battery-Aware Routing (BAR) [20], each node can forward data toward the sink node based on a multi-hop routing computation that maximizes the total energy of a route. In [21], a routing protocol similar to the AODV [22] is proposed. In this solution, called Energy-Aware Routing Protocol (EARP), a node that needs to send data starts a flooding process that is maintained until the packet reaches the destination. However, differently from AODV, EARP can estimate better route expiration times based on the residual energy of the nodes, resulting in better energy usage of the nodes. GAF (Geographic Adaptive Fidelity) [23] works by dividing the sensor area in a grid and uses the GPS coordinate of a node to estimate in which cell it is located. After that, nodes can be divided into three states: discovered, awake, and sleeping so that only a single node remains awake in a cell while the other nodes in this cell are sleeping. The generated grid is computed in such a way that every node in a cell is able to communicate to the other nodes in neighboring cells. By doing this, the algorithm guarantees the existence of routes toward the sink node. Finally, energy awareness has also been applied to other WSNs algorithms such as coverage control [20] and clusters formation [24,25,26,27].

In the third category, the algorithms are based on the capability of the sensor nodes to convert available environmental energy to electrical energy (e.g., solar cells) so that the battery of the sensor nodes can be recharged. In [28], the authors propose an algorithm that computes the best path between source and target based on the energy by using an on-demand routing. In [29], two greedy forward routing protocols are proposed based on a model that chooses the best routes based on the local information of neighbors, thus resulting in an increased lifetime of the network. In Adaptive Energy Harvesting Aware Clustering (AEHAC) [30], a cluster-based algorithm that uses the battery and energy harvesting information of the nodes is proposed. This information is used to elect the cluster-heads (CH), which will be the nodes with greater energy availability. Another cluster-based algorithm is proposed in [31]. The proposed solution elects new cluster-heads periodically, but only after reserving energy for them to avoid the energy-shortage problem. Similarly, the Energy Harvesting Opportunistic Routing protocol (EHOR) [32] is an opportunistic routing algorithm that uses the residual energy of the nodes to choose which node will be used to forward the data. More recently, in [33], a routing algorithm was proposed for rechargeable wireless sensor networks in which fixed optimal routing paths for data delivery are not maintained and, instead, data traffic flows from a sensor to its neighbors freely to save energy.

Regarding our proposed REBORN algorithm, we can say that it has characteristics from all three categories. Our algorithm can make routing decisions based on the residual energy information from the nodes. Furthermore, each node is able to change its own state based on its energy consumption and availability. Finally, our solution considers that each node is equipped with a small solar cell so that it can collect and store energy. We can also say that our solution has some aspects from GAF, since it uses a similar grid scheme, and has some aspect from EHOR since it uses an opportunistic routing scheme to forward data toward the sink node.

## 3. REBORN-Renewable Energy-Based Routing Algorithm

In this section, we present our proposed REBORN (Renewable Energy-Based Routing) algorithm. As mentioned before, our algorithm assumes that each sensor node is equipped with a small solar cell, allowing them to recharge their batteries according to the available solar intensity (that varies during the day/night cycle). Different from most proposed solutions, REBORN uses both the residual battery level and the intensity of the available energy to decide on whether to send and/or forward data.

**Figure 1:**
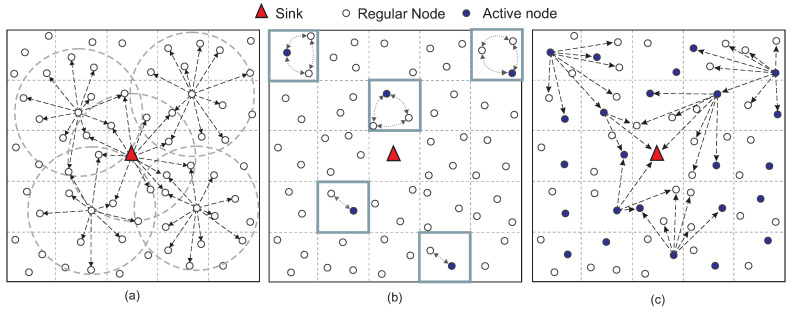
REBORN example: (**a**) the sink node starts a controlled flooding to send its position information to the network; (**b**) the nodes in each cell, which are in the discovery state, elect the node that will remain active; (**c**) an active node sends its data by broadcast, and active nodes in adjacent cells opportunistically forward the data toward the sink node using a greedy forwarding scheme.

REBORN, as shown in Algorithm 1, starts when the sink node initiates a controlled flood (Figure 1a). In this packet, a routing message containing its position in the grid is sent (line 11). To control the flooding, a variable that determines whether the packet was received by the node is maintained (lines 13 and 14). If the node has not yet received the packet, it computes its distance to the sink and forwards the message to its neighbors (lines 15 and 16); otherwise, the node ignores the duplicated packet. The node then computes the grid it belongs to by using its known position (line 17). This process continues until all of the network nodes receive the sink data.
**Algorithm 1:** REBORN▷ **Variables:**1:  $aTimer_{i}$;{Time spent in activity state}2:  $eTimer_{i}$;{Time spent in energy-saving state}3:  $discTimer_{i}$;{Time spent in the discovery state}4:  $remTimeAct_{i}$;{Remaining time for the node to exit the active state}5:  state := discovery;{Current node state}6:  $routeConf_{i}$ := **false**;{Routing message received}7:  $sinkDist_{i}$;{Distance to the sink}8:  $gridID_{i}$;{Identification of the cell}9:  $msg_{i}$;{Identifier of incoming data messages}▷ **Input:**10:  Sink node sends routing message **Action:**11:  $sendRouteMsg(sinkPos_{i})$ {Sink initiates a controlled flooding}▷ **Input:**12:  Regular nodes receive routing message (msg${}_{Route}$) **Action:**13:  **if** !routeConf${}_{i}$ **then**{Was message received?}14:   routeConf${}_{i}$ := **true**;{Confirmed message}15:   sinkDist${}_{i}$ := getDist(msg${}_{Route}$.sinkPos${}_{i}$);{Compute distance of sink}16:   $sendRouteMsg(sinkPos_{i})$;{Forward the message}17:   gridID := getGrid(nodePos);{Compute the grid ID}18:  **end if**▷ **Input:**19:  Regular nodes enter in discovery state **Action**20:  discTimer${}_{i}$ := *Constant*{Update discTimer${}_{i}$ value}21:  aTimer${}_{i}$ := remTimerAct${}_{i}$ := getTimeToSend();{Compute the send time}22:  timer${}_{discTimer_{i}}$.trigger(){Time to node change the state}23:  sendDiscMessage(nodeID, gridID, remTimeAct, state, energyBat);▷ **Input:**24:  Regular nodes receive discovery message (msg${}_{Dis}$) **Action**25:  **if** state == discovery **and** msg${}_{Dis}$.state == active **then**{High priority?}26:   eTimer${}_{i}$ := msg${}_{Dis}$.remTimeAct; state := sleep;27:  **else if** state == msg${}_{Dis}$.state **then** {Are nodes in same state ?}28:   **if** energyBat < msg${}_{Dis}$.energyBat **then**{Is my energy bigger ?}29:    eTimer := msg${}_{Dis}$.remTimeAct; state := sleep;30:   **end if**31:  **end if**▷ **Input:**32:  Timer${}_{discTimer_{i}}$ fire **Action**33:  state := active{The elected node changes to active state}▷ **Input:**34:  Elected nodes enter in active state **Action**35:  discTimer${}_{i}$ := aTimer${}_{i}$/6;{Update discTimer${}_{i}$ value}36:  Timer${}_{aTimer_{i}}$.trigger();{Time to node change state}37:  Timer${}_{aTimer_{i}/2}$.trigger();{Time to node send the data message}38:  Timer${}_{DiscTimer_{i}}$.trigger();{Time for node to send the discovery message}▷ **Input:**39:  Timer${}_{DiscTimer_{i}}$ fire **Action:**40:  remTimeAct${}_{i}$ := timer${}_{aTimer}$.remainingTime();{Update remTimeAct${}_{i}$ with rest time of aTimer${}_{i}$}41:  sendDiscMessage(nodeID, gridID, remTimeAct, state, energyBat);▷ **Input:**42:  Timer${}_{aTimer_{i}/2}$ fire **Action:**43:  sendDataMessage(data, msg${}_{i}$, sinkDist);▷ **Input:**44:  Nodes in active state receive data message (msg${}_{Dat}$) **Action:**45:  **if** sinkDist${}_{i}$ < msg${}_{Dat}.sinkDist_{i}$**and** !checkID($msg._{Dat}.msg_{i}$) **then**46:   sendDataMessage(msg${}_{Dat}$.date, msg.${}_{Dat}$.msg${}_{i}$, distSink);47:   msg${}_{i}$.add(msg.${}_{Dat}$.$msg_{i}$);{Storage msg${}_{i}$}48:  **end if**▷ **Input:**49:  Nodes not elected enter in the sleep state **Action:**50:  timer${}_{eTimer_{i}}$.trigger();{Time to node change state}▷ **Input:**51:  Timer${}_{aTimer}$ or Timer${}_{eTimer_{i}}$ fire **Action:**52:  state := discovery;{Restart the process}

After the configuration, the nodes go into the discovery state. In this step, the algorithm starts a first timer ($discTime_{i}$) that will be used to identify the time the node will remain in the discovery state (line 20). A second timer, which will be used to control the time a node will remain active, is also initialized (line 21). Such a timer is configured based on both the current solar intensity and the amount of remaining battery power (detailed in the next sections). At this point, a control variable called $remTimeAct_{i}$ will be used to store the remaining uptime on each node. When the first timer is started, the node broadcasts a message to all of its neighbors located in the same cell (line 23), as depicted in Figure 1b.

In the discovery message submission step, nodes in the same cell exchange information with each other. This exchange of discovery messages among nodes enables the switching process between active and energy-saving modes, used to elect the neighbor responsible for forwarding messages toward the sink node. To establish a higher level of control during the message exchange process, some rules are proposed. A node that has the active state has higher priority so that, if a node is in the discovery state and it receives a message from a node that is in the active state (line 25), this node will immediately switch to sleep mode (line 26). The node in the active state coordinates the time duration of the node in the sleep mode, in such a way that the nodes enter discovery mode at the same time. On the other hand, if the nodes are in the same state (line 27), the remaining energy is checked (line 28). This node will switch to energy saving mode (line 29) if it receives a message from a neighbor who has more energy. In this case, if the nodes are in the active state, the rule is applied immediately, but if they are in a state of discovery, they will be able to receive the information of the neighbors so that they know which neighbor has the largest amount of energy. Finally, when the first timer expires, the state change happens (line 33).

When a node is in the active state, the information stored in the variable $discTimer_{i}$ is updated (line 35). From this point on, a new set of helper timers will be needed for the management of power modes (lines 36–38). While the node remains in the active state, it will be possible to send discovery packets at $discTimer_{i}$ time intervals, that is, every time the first timer expires, the value of $remTimeAct_{i}$ is updated with the time the node will remain active (lines 40 and 41). If $remTimeAct_{i}$ is greater than $discTimer_{i}$, the timer in question will be re-enabled. When the helper timer $aTimer_{i}/2$ expires, a broadcast message is sent to the network (Figure 1c) containing a data packet with the gathered data, the message identifier, and the distance to the sink (line 43). Finally, when the helper timer $aTimer_{i}$ expires, the node returns to the discovery state (line 52). During the process of forwarding packets to the sink, if a node is in the active state and receiving a packet, it checks if the received message came from a node farthest from the sink. In addition, the node checks if it has already received this message previously (line 45), and if it has received it, checks if the sender is a node farthest from the sink. If the node has not yet received such a message, the node updates its distance information and prepares a message with its distance to the sink. This message will then be broadcasted (lines 46 and 47), otherwise, the received packet will be discarded.

**Figure 2:**
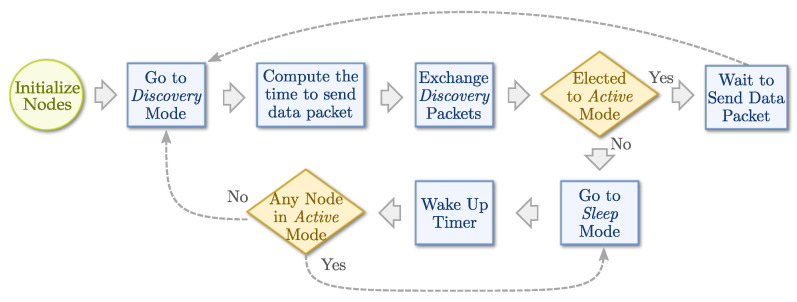
REBORN flowchart of states and decisions executed by each node.

When a node enters the save state, all timers already configured are deactivated. However, it is possible for the node to switch to a more battery-saving mode. In these cases, the timer configured upon receipt of a discovery message will be activated (line 50). Finally, when this timer expires, the node returns to discovery mode (line 52) and the election process is restarted. To better visualize all of these steps and state changes, Figure 2 shows a flowchart of states and decisions for the nodes.

### 3.1. Renewable Energy

To evaluate the performance of our routing algorithm, we need a way for simulating the harvested energy. Some solar models can be found in the literature [34,35] that focus on being the most robust and realistic, taking into consideration aspects such as seasonal variations, weather forecasts, and even the dissipation of energy by voltage regulators. However, these models are complex and provide an unnecessary level of detail for our case. Thus, based on more simplistic but still realistic models [36], in this section, we describe our proposed solar model.

**Figure 3:**
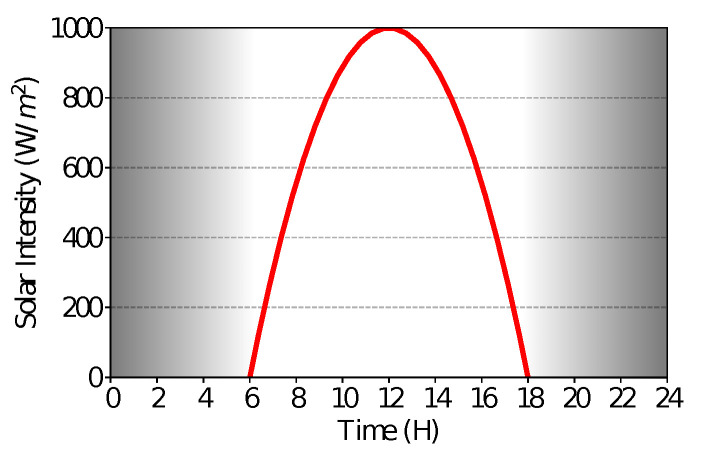
Theoretical model of solar intensity.

The first step is to understand the behavior of the solar intensity. As shown in [37], the light intensity begins approximately at 06:00, grows up to noon, and decreases until 18:00. To model this behavior, we used a quadratic function fit with three hour/solar-intensity points: (6, 0), (12, 1000), and (18, 0). These points were used to find the coefficients of the function, shown in Equation (1). The value 1000 at noon was used because of the Watt-pico (Wp), a common measure used in solar panels, to represent the maximum solar intensity input.

(1) $$\phi =-27.778\chi^{2}+666.667\chi -3000.024$$

where χ is the time measured in hours. As we can see in Figure 3, the model has a maximum intensity of 1000 W/m${}^{2}$ around noon and no intensity during the night.

To determine how much energy is generated by Equation (1), a model for the solar panel is also created. Considering no loss by the conversion of energy, the output power of the solar panel is described by Equation (2).

(2) $$\Psi =\frac{\gamma \phi }{\Lambda }$$

where Ψ is the power output, γ is the maximum output of the solar panel, ϕ is the current solar intensity, and Λ is the maximum solar intensity, as detailed in Table 1.

From Equations (1) and (2), we can compute the energy generated considering periods of 1 second between 06:00 and 18:00. For each second, we can compute the solar intensity using Equation (1) and use the result in Equation (2). At 18:00, the accumulated energy generated was 4319.5 J.

**Figure 4:**
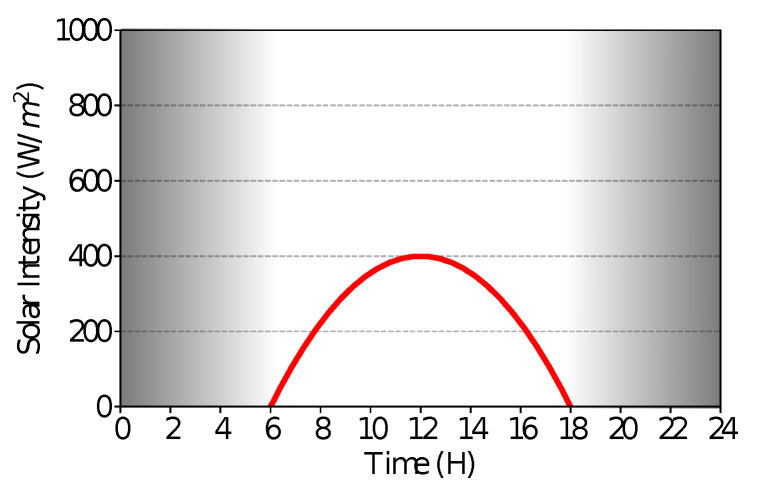
Adjusted model of solar intensity.

However, this energy generated is based on a very optimistic behavior from experiments made in laboratories. As shown by some studies [38], we can see that in a real-world scenario, a small, sensor-based solar panel of 0.15 W can only generate a total energy of 3240 J. However, even this case is an optimistic one, since nodes in WSNs will be located, in most cases, in places with lower solar intensity (e.g., forests) or in places where the lightness does not reach directly or at a good angle. Thus, we reduce the accumulated energy even more to about 1727.8 J by changing Equation (1) to reduce the solar intensity by a constant value of 0.4. Finally, Equation (3) shows the final model used by our simulations while Figure 4 shows its respective curve.

(3) $$\phi =(-27.778\chi^{2}+666.667\chi -3000.024)*0.4$$

### 3.2. GAF-EH

Our proposed solution is based on the GAF algorithm, introduced in [23]. An improvement was implemented so that the approach would also be able to collect renewable energy. Considering the energy expenditure observed when a node is in listening mode, mechanisms to reduce energy consumption from the nodes were implemented. In order to provide a fair comparison among the approaches proposed in this work, we adapted the GAF algorithm so that it was possible to collect energy. Based on this adaptation, it was considered that if the node’s battery was completely depleted, the node would be able to recharge the attached battery and, at the same time, be able to operate in power-saving mode.

### 3.3. Control Mechanisms

In order to better manage the energy model proposed in this work, we implemented a packet sending control mechanism that is based on both the solar intensity received and also on the amount of energy available in the battery so that the interval between the data packets had a direct relationship with these variables. The main idea was that if the node had a lower residual battery, fewer packets would be sent. In this sense, the same principle was adopted for the solar intensity, that is, lower solar intensities would reduce the number of packets to be sent.

#### 3.3.1. Battery Management

**Figure 5:**
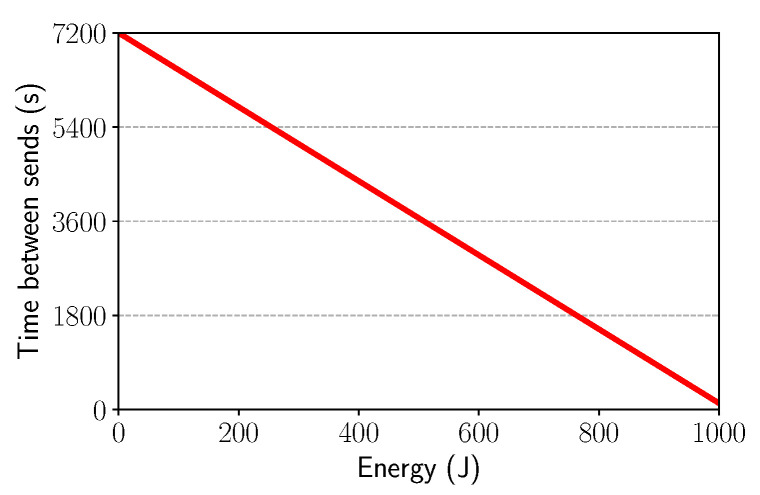
Battery power level vs. time.

Based on the control mechanism discussed previously, we considered that the data packet interval would increase as the amount of battery power decreases. Thus, when the energy was close to zero, the number of packets would be as small as possible. In this way, energy-aware is characterized, since the amount of packets sent is related to the amount of energy available in the battery. Thus, we need a way to compute the time to send the next data packet for each sensor node. As mentioned, this time needs to be related to the battery level of the node. Equation (4) is responsible for this behavior:

(4) $$\omega =\left(\alpha -\varphi \right)\left(\frac{\beta -\delta }{\sigma -\delta }\right)+\varphi$$

whose variables and default values are described in Table 2. This equation was derived from the classic line equation passing throw the points (σ, α) and (δ, φ), which are the worst and the best cases, respectively. As depicted in Figure 5, the time for sending data packets decreases (i.e., more packets are sent) as the energy level of the node increases.

#### 3.3.2. Solar Intensity Control

**Figure 6:**
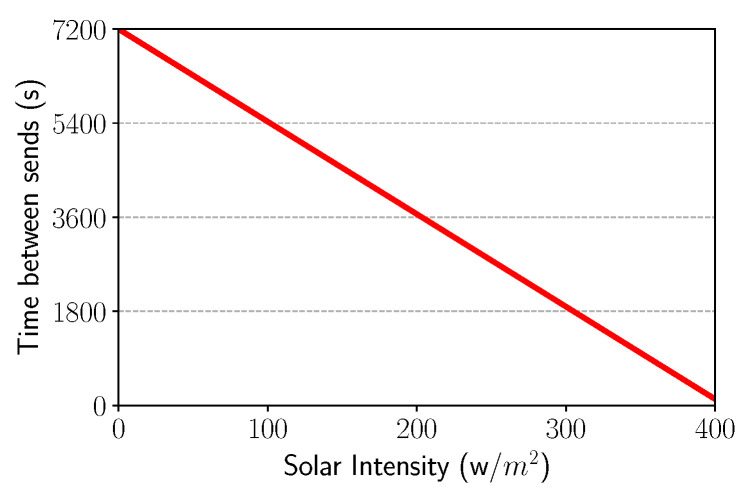
Solar intensity vs. Time.

Similar to the battery management control, when solar intensity is low or nonexistent, the interval between packet sending should be as large as possible since the battery is not being recharged. Thus, the node has enough information about the energy collection, characterizing the awareness of energy harvesting. Equation (5) is responsible for this behavior and Table 3 describes the variables and values used in the simulation, as depicted in Figure 6. The Equation (5) was derived by line equation form based at these two points (ρ, α) and (λ, φ), which are the worst case and best case, respectively.

(5) $$\Omega =\;\left(\alpha -\varphi \right)\left(\frac{\phi -\lambda }{\rho -\lambda }\right)\;+\;\varphi$$

It is important to note that Equations (4) and (5), considers a linear relationship between the number of packets to send and both the battery level and the solar intensity. Other curves (e.g., parabola or a third-degree curve) could result in different and interesting relationships. For instance, a logarithmic curve could result in more drastic behavior such as almost stopping sending packets even when the node still has some energy available. This kind of behavior could be useful in some applications. We intend to propose and evaluate the performance of different curves in future work.

#### 3.3.3. Battery + Solar Intensity

**Figure 7:**
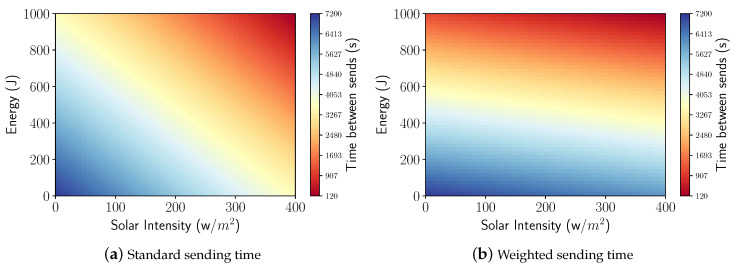
Relation between packet sending times.

In order for the two control mechanisms to work together, an average was provided between Equations (4) and (5), allowing the creation of a single model that effectively expressed both battery energy and solar intensity. This model is shown in Equation (6). Therefore, when using the standard values of the simulations, we have the corresponding packet intervals depicted in Figure 7a.

(6) $$\mu =\frac{\left(\alpha -\varphi \right)\left(\frac{\beta -\delta }{\sigma -\delta }+\frac{\phi -\lambda }{\rho -\lambda }\right)+2\varphi }{2}$$

To control how both the charge level of the battery and the solar intensity are perceived during the harvesting process, a parameter *K* was adopted to give more freedom to the algorithm and manage the packets sent with the priority of choice between the solar intensity and battery energy. This parameter is a configuration value that can be set during the network initialization or changed during operation. All nodes have the same *K* value that can vary from 0 to 1 and is associated with battery usage. For instance, a value of K=0.6 assumes that the node will wait a while before sending its information, which corresponds to 60% of the remaining time of the battery charge, added to 40% of the time of the solar intensity perceived. Finally, the final version of our model is shown in Equation (7).

(7) $$\mu =\left(\alpha -\varphi \right)\left[\left(\frac{\beta -\delta }{\sigma -\delta }\right)K+\left(\frac{\phi -\lambda }{\rho -\lambda }\right)\left(1-K\right)\right]+\varphi$$

By using a value of K=0.85 and the values adopted in our simulations, the corresponding time intervals are depicted in Figure 7b, implying that the battery remaining has more importance than solar intensity.

## 4. Performance Evaluation

In this section, we evaluate the performance of our proposed REBORN algorithm and compare it to three other algorithms. The first of the three algorithms used as a basis for comparison was the GAF-EH (explained in Section 3.2). This algorithm does not have any type of packet-sending control mechanism. The second is the REBORN-BAT, which uses only the residual energy information of the node (as described in Section 3.3.1). The third one, called REBORN-SI, is based only on the solar intensity information perceived in the environment (as described in Section 3.3.2). Finally, the last algorithm, named REBORN-both, is our proposed solution that uses both residual energy and solar intensity (as shown in Section 3.3.3). The main reason to compare our final REBORN algorithm to both REBORN-BAT and REBORN-SI is to better understand the impact of each part of the algorithm. In all algorithms, the minimum interval between data packet transmissions to the network was 2 min.

With the exception of GAF-EH, all algorithms were able to send their packets at intervals of no more than 2 h. In this sense, if the battery had a high charge level and the solar intensity on a high level, the data would be sent every 2 min. Furthermore, if the solar intensity was weak or nonexistent and the battery level was low, the node would send its data every 2 h.

### 4.1. Methodology

The simulations were carried out using the Sinalgo simulator [39], which provides a complete environment for the simulation of distributed algorithms. We assumed that each simulation round corresponds to 1 s for a total of 72 h (i.e., three days of simulation). The dawn of a new day began at 06:00 a.m. while dusk was at 06:00 p.m. This assumption is relevant because it defines the beginning and end of the energy harvest each day. The algorithm was set to start running at 00:00 a.m., and end at 11:59 p.m. on the third day.

The maximum battery charge of the nodes at the beginning of each simulation was 1000 Joules. The resident load was depleting as the rounds were executed until the designed end of the simulation. When the simulation reached the time corresponding to 06:00 a.m., the energy harvesting mechanism from the solar panel was started, lasting until 06:00 p.m. This harvesting process was repeated in the following two days. Each node was equipped with a GPS device. Furthermore, the nodes were aware of the amount of energy residing in their batteries, just as if they were aware of the solar intensity to which they were subjected. The sensing area was 132 × 132 m${}^{2}$, keeping the node density in 0.03 nodes/m${}^{2}$ and obeying a disturbed grid.

Regarding the results, curves represent average values, while error bars represent confidence intervals for 95% of confidence from 33 different random seeds. The simulation parameters are based in [40] and shown in Table 4.

### 4.2. Energy Consumption

**Figure 8:**
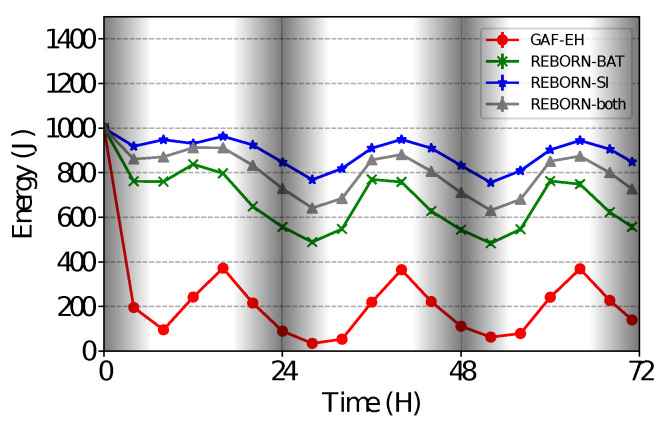
Energy consumption over time.

Figure 8 shows the average energy consumption during the 3 days of simulation. As can be seen, the GAF-EH received the lowest score among all, since there is no type of management in sending packets with regard to energy expended. The results also show that the REBORN-BAT algorithm, which has the control based on the battery level, the energy saving is expressive in relation to the algorithm that does not have any control. On the other hand, the energy consumption observed in the execution of the REBORN-SI algorithm is lower than that observed in all others. This is due to the fact that the amount of packets sent during the night is the minimum possible due to the energy management controls. Furthermore, REBORN-SI is able to modify the intervals for sending packets according to the solar intensity, that is, the packets will be sent regularly only during the period of energy harvest. Finally, these results show that our REBORN-both algorithm was able to get the best of both cases, i.e., to send more data when energy is abundant and save energy in other cases.

### 4.3. Impact of the Dead Nodes

**Figure 9:**
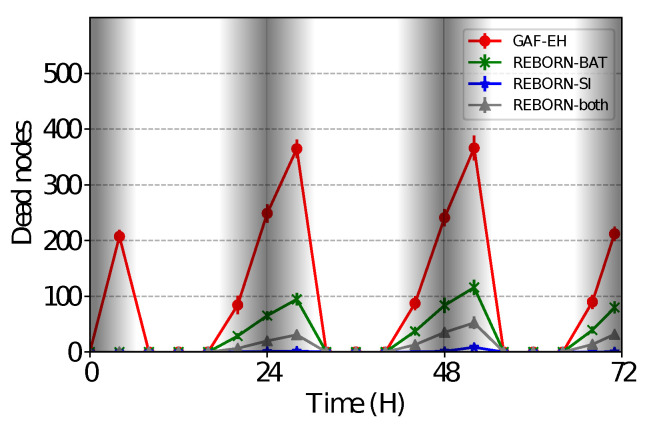
Number of dead nodes over time.

As shown in Figure 9, it is possible to see that the GAF-EH algorithm results in a greater number of dead nodes (i.e., nodes without remaining energy), due to its absence of control when sending data to the sink node. At this point, two considerations are necessary. First is that the energy of the nodes next to the sink is depleted more quickly because of a large number of exchanged packets, as it is possible to observe in the shaded area of the mentioned figure. The second consideration is that during the diurnal cycle, the amount of energy expended is greater than the harvested energy.

### 4.4. Delivery Rate

**Figure 10:**
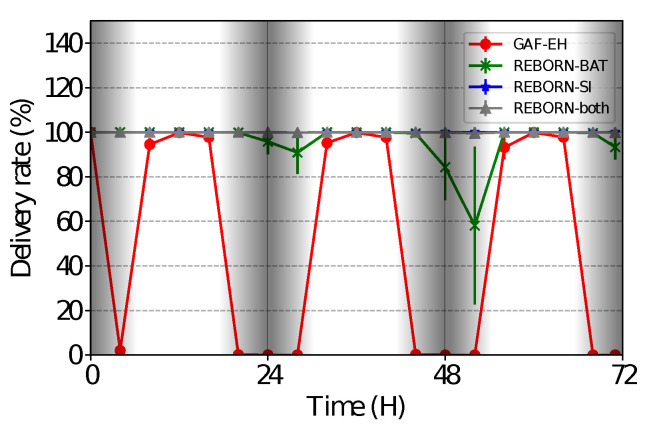
Delivery rate of sent packets.

As shown in Figure 10, it is possible to observe that in the REBORN-SI algorithm, there are periods of inactivity perceived during the first two nights of simulation. This is due to the increase in the time interval between shipments, which in this sense occurs every 2 h, making it impossible to deliver packets to the sink during these periods of inactivity. Although REBORN-both has a greater number of dead nodes than REBORN-SI, we can see that the delivery rate has not been affected. In this sense, we observed the effectiveness of the controls implemented in our proposed algorithm. Finally, it is possible to observe that a relevant amount of packets to be forwarded by the GAF-EH algorithm do not reach the sink. This behavior was already expected, since the algorithm does not have energy economy controls.

### 4.5. Sent Packets

**Figure 11:**
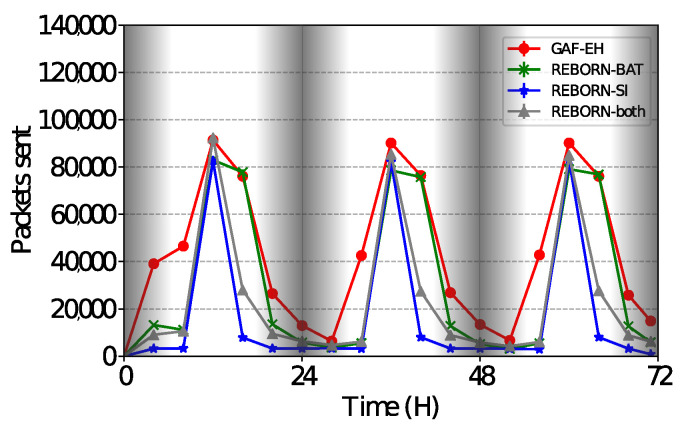
Number of sent packets over 3 days.

In the present results, we evaluated the number of packets sent during the simulations. As can be seen in Figure 11, the GAF-EH algorithm presents significant variations in the sending rates. This is due to the natural energy depletion of the nodes and consequently the process of harvesting energy from the nodes. As expected, this algorithm sends a large number of packets, although it has already been shown to be infinitely related to the lifetime (activity). However, the REBORN-BAT algorithm sends smaller quantities of packets, even though both the number of dead nodes and the delivery rate were found to be unsatisfactory.

The smallest number of packets observed among the evaluated algorithms is that of REBORN-SI. This is covered by managing the amount of energy already stored despite having its focus on power consumption while sending packets.

**Figure 12:**
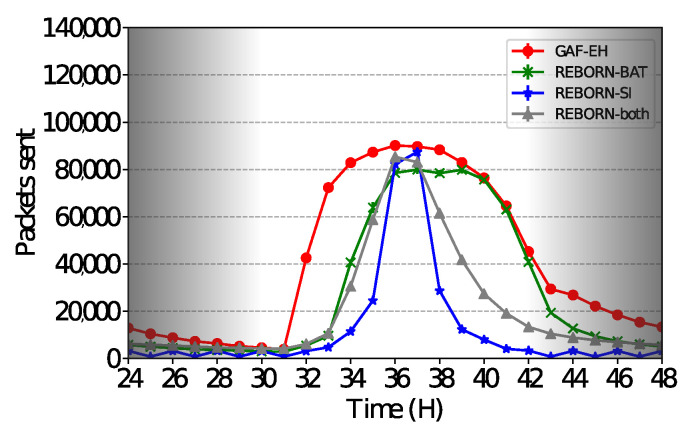
Number of sent packets over 1 day.

Regarding REBORN-SI, REBORN-both sends a larger number of packets although both have similar delivery rates. Based on these evaluations, it is possible to state that REBORN-both can send larger quantities of packets to the sink and still maintain high rates of delivery. The relation between cost and benefit of using the REBORN-both algorithm can be adjusted through the parameter K, as observed in Equation (7). Figure 12 shows details of the simulation performed between 24 and 48 h.

### 4.6. Impact of the Network Density

**Figure 13:**
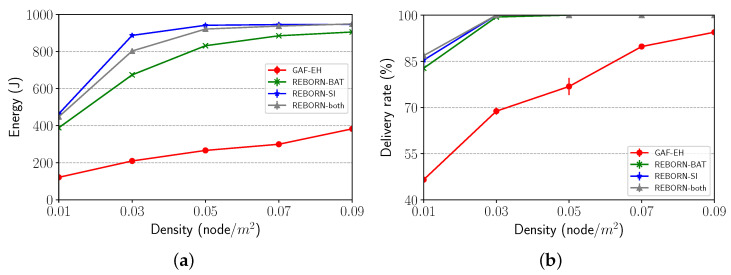
Impact of density on (**a**) energy consumption and (**b**) delivery rate.

The impact of the network density was evaluated by increasing this metric from 0.01 to 0.09 nodes/m${}^{2}$. As shown in Figure 13a, despite the high density, the GAF-EH still kept its residual energy low, when compared to the other algorithms. On the other hand, it is possible to observe in Figure 13b that the delivery rate for all solutions increases when the density of nodes increases.

### 4.7. The Impact of Scalability

**Figure 14:**
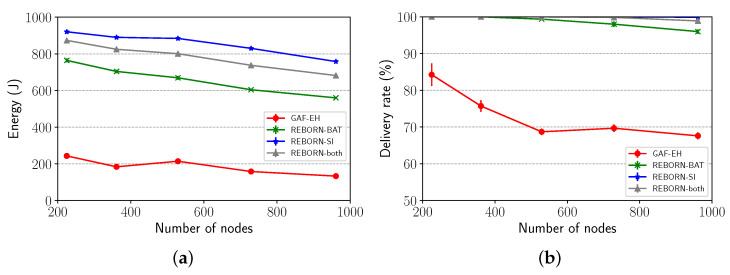
Impact of scalability on (**a**) energy consumption and (**b**) delivery rate.

Finally, the scalability was evaluated by increasing the network size from 225 to 961 nodes with a constant density of 0.03 nodes/m${}^{2}$. The grid in which the sensors were implanted was resized according to the number of sensor nodes. As can be seen from Figure 14a, energy consumption tends to increase as the number of nodes in the grid increases. In this sense, as the density value holds the same, more cells are generated in the grid. Because of the increase in the number of cells in the grid, the number of packets to be forwarded also increases, according to the position of the nodes in relation to the sink, that is, the closer the node is to the sink, the more packets it will go forward. In this way, a greater amount of energy will be spent in the referral process. Finally, despite the observed energy cost, REBORN-both shows scalable because it presents a satisfactory delivery rate even when the number of cells in the grid increases.

## 5. Conclusions

In this work, we proposed a new routing protocol, called REBORN (Renewable Energy-Based Routing), that takes advantage of both the residual battery energy and the available renewable energy to allow energy savings in the wake/sleep schedules of the nodes and also on the management of the number of data packets sent to the sink node. Our proposed approach uses a grid-based opportunistic geographic routing to forward data packets between cells toward the sink node.

Extensive simulations were carried out in several scenarios to evaluate the performance of the proposed solution. We also compared our solution to a variation of the GAF algorithm that uses energy harvesting. The results show that our proposed control mechanism can maintain a good cost/benefit relationship regarding the energy usage and the number of data packets sent.

The results are very promising, but some advantages and limitations still need to be further explored in future works. For instance, we intend to improve the current energy model as well as our proposed solution so they can adapt to seasonal variations in sunlight and diurnal cycles and also take into consideration the weather forecast for the next few days to make better decisions on whether to save energy (on cloudy days) or to spend energy sending more data (on sunny days). Finally, as discussed in Section 3.3.2, we also intend to propose and evaluate the performance of different behavior curves in future work.

## Figures and Tables

**Table 1:** Solar panel model variables.

Variable	Standard Value	Function	Unit
Ψ	-	Current output power of solar panel	W
γ	0.150	Maximum output power of solar panel	W
Λ	1000	Maximum solar intensity possible on the solar panel	W_P_
ϕ	–	Current solar intensity in the solar panel	W/m^2^

**Table 2:** Battery management equation variables.

Variable	Default Value	Function	Unit
α	7200	Maximum interval between sending out packets	s
φ	120	Minimum interval between sending out packets	s
δ	1000	Maximum battery-resident energy	J
σ	0	Energy threshold to arrive at α	J
β	–	Current battery charge level	J
ω	–	Time to send the next packet	s

**Table 3:** Solar intensity equation variables.

Variable	Standard Value	Function	Unit
α	7200	Maximum interval between sending out packets	s
φ	120	Minimum interval between sending out packets	s
λ	400	Maximum solar intensity chosen	W/m^2^
ρ	0	Solar intensity threshold to arrive at α	W/m^2^
ϕ	–	Current solar intensity in solar panel	W/m^2^
Ω	–	Time to send the next packet	*s*

**Table 4:** Default simulation scenario.

Parameters	Standard Value
Density	0.03 nodes/m^2^
Sink Position	Center of the monitored area
Monitored area	132 × 132 m^2^
Number of nodes	529 nodes (disturbed grid)
Communication range	30 m
GPS inaccuracy	3 m
Energy spent to send packets	0.11385 J
Energy spent on receiving packets	0.05973 J
Energy spent in idle mode	0.01716 J
Energy spent in sleep mode	0.00099 J
Initial residual energy of the nodes	1000 J

## Data Availability

Not applicable.
